# Endovascular Repair of Abdominal Aortic Aneurysms is a Valid Alternative to Open Repair also in Patients Treated Outside of Instructions for Use Criteria

**DOI:** 10.1007/s00270-022-03297-7

**Published:** 2022-11-04

**Authors:** Sara Protto, Tilda Hahl, Kalle J. A. Koskinen, Valtteri Järvenpää, Ilkka Uurto, Suvi Väärämäki, Velipekka Suominen

**Affiliations:** 1 Department of Vascular and Interventional Radiology, Tampere University Hospital, P.O. BOX 2000, 33521 Tampere, Finland; 2 Tampere University, 33014 Tampere, Finland

**Keywords:** Abdominal aneurysm, Instruction for use, Endovascular repair, Open surgical repair, Endograft

## Abstract

**Purpose:**

It remains unclear whether endovascular aneurysm repair, in the long term, is less effective than open surgery due to need for reinterventions and close monitoring. We aimed to evaluate this matter in a real-life cohort.

**Methods:**

We collected consecutive patients treated with EVAR or OSR between January 2005 and December 2013. Primary outcomes were 30-day, 90-day and long-term all-cause mortality. Secondary outcomes were 30-day reintervention rate and reintervention-free survival. We evaluated also a subpopulation who did not adhere to IFU.

**Results:**

The inclusion criteria were met by 416 patients. 258 (62%) received EVAR, while 158 (38%) underwent OSR. The 30- or 90-day mortality was similar between groups (*p* = 0.272 and *p* = 0.346), as ARM (*p* = 0.652). The 30-day reintervention rate was higher in the OSR group (*p* < 0.001), but during follow-up, it was significantly higher in the EVAR group (log-rank: 0.026).

There were 114 (44.2%) non-IFU patients in the EVAR group, and we compared them with OSR group. There was no significant difference in all-cause mortality at 30 or 90 days, nor in the long term (*p* = 1; *p* = 1 and *p* = 0.062). ARM was not affected by the procedure technique (*p* = 0.136). The short-term reintervention rate was higher in the OSR group (*p* = 0.003), while in the long-term EVAR, patients experienced more reinterventions (log-rank = 0.0.43).

**Conclusion:**

No significant difference in survival was found between EVAR and OSR, independent of adherence to IFU. EVAR may be considered for surgical candidates.

**Supplementary Information:**

The online version contains supplementary material available at 10.1007/s00270-022-03297-7.

## Introduction

Endovascular aneurysm repair (EVAR) has become a well-established option for the treatment of abdominal aortic aneurysms (AAA) after its introduction. A few randomized controlled trials (RCT) have compared EVAR and open surgical repair (OSR), showing that while the short-term overall survival is better in the endovascular group, this advantage is lost within 3 years [1–4]. Moreover, two of these studies demonstrated an increasing rate of secondary procedures during follow-up in the endovascular group [1, 2]. Recently, the UK’s National Institute for Health and Care Excellence (NICE) has proposed that open surgical repair should be offered for patients with unruptured AAAs “unless it is contraindicated because of their abdominal copathology, anesthetic risks and/or medical comorbidities” [5], while in real life, EVAR is the most common treatment method for an AAA [6].

It remains unclear whether endovascular repair is, in the long term, less effective than open surgery due to a higher rate of reinterventions, morbidity and mortality, as well as the consequent need for close monitoring. The RCTs patients met strict selection criteria; however, in real life, 40%–44% of the patients are treated outside of the manufacturers’ instructions for use (IFU), even though the effect of non-adherence to IFU is controversial [7–9]. Consequently, it is unclear which treatment option should be preferred in the case of non-adherence to IFU. Often EVAR is recommended to avoid, in the short term, the potential impact of complications of open procedures in patients thought to be at higher risk. The study by Charbonneau et al. is the only paper we found addressing this issue. According to their findings, it seems that open repair should be preferred in the case of non-adherence to IFU [10].

The objective of this study was to compare endovascular and open surgical repair of AAAs in a real-life cohort and, further, to evaluate which treatment modality should be offered in case adherence to the IFU for EVAR is not possible due to angioanatomical features.

## Materials and Methods

We identified 544 consecutive patients who were treated electively with either EVAR or OSR for an infrarenal AAA between January 2005 and December 2013 at our institution. The exclusion criteria were: the absence of pre-operative computed tomography angiography (CTA), an isolated iliac aneurysm, or a ruptured aneurysm. Urgently managed patients with symptomatic or massive aneurysms were included.

Patients were eligible for either treatment modality if they presented with an AAA diameter of > 5.5 cm (male) or > 5.0 cm (female), or an AAA with a rapidly increasing sac (> 1 cm per year or > 5 mm over a 6-month period). The treatment decision was made at the discretion of the treating surgeons and interventional radiologists, during the multidisciplinary team (MDT) meeting.

The EVAR procedures were performed in a fully equipped operating theatre with fluoroscopic guidance. EVAR patients were treated percutaneously under spinal or general anaesthesia. In the case of OSR, the procedure was performed under general anaesthesia following standard surgical protocols for aortic surgery.

The anatomic measurements were obtained from CTA using axial, sagittal and coronal views and multi-planar reconstructions (MPR). Aortic neck diameter, length, angulation and tapering, as well as aneurysm diameter, common iliac length and diameter, and external iliac diameter were collected.

For the subgroup analysis regarding IFU adherence, we defined patients in the EVAR group as “non-IFU”, if at least one anatomical parameter violated the manufacturer’s criteria for that specific device (Table 1); moreover, we evaluated neck calcification and neck conicity. A conical neck shape was defined as in the ESVS guidelines: over 3 mm increase in neck diameter for each centimetre of length [11]. A relevant proximal neck thrombus was defined as a ≥ 50% circumferential thrombus and proximal neck calcification as ≥ 50% calcification.

**Table 1:** Device-specific anatomic instructions for use (IFU) criteria and type of endografts and prothesis used

Anatomic criteria	Medtronic	Gore	Zenith
Proximal aortic neck length, mm	≥ 10	≥ 15	≥ 15
Proximal aortic neck diameter, mm	19–32	19–29	18–32
Proximal aortic neck angulation, degrees	≤ 60	≤ 60	≤ 60
Common iliac artery length, mm	≥ 15	≥ 10	> 10
Common iliac artery diameter, mm	8–25	8–25	7.5–20
External iliac artery diameter, mm	8–25	8–25	7.5–25
Endograft used	Zenith (Cook, Bloomington, IN, USA), Endurant (Medtronic, Minneapolis, MN, USA) Excluder (W.L. Gore, Flagstaff, AZ, USA)		
Prothesis	Dacron-coated prothesis (B. Braun, Berlin, Germany, Terumo, Boston Scientific) PTFE graft (W.L. Gore, Flagstaff, AZ, USA)		

Baseline demographic data were recorded, including sex, age and comorbidities (Table 2).

**Table 2:** Main population baseline characteristics

	All		
	EVAR	OSR	*p*
	*N* = 258	*N* = 158	
Age, mean (years) SD	76.4	68.8	**0.000**
	SD 7.3	SD 8.5	
Sex, male %	85.3	89.2	0.246
non-IFU %	36	39.9	0.434
Hypertension %	67.1	67.7	0.888
Dyslipidemia %	45.7	45.6	0.974
Coronary artery disease %	49.2	31	**0.000**
Diabetes %	18.6	17.1	0.696
Cerebrovascular disease %	14.3	9.5	0.147
Pulmonal disease %	22.1	17.7	0.283
Peripheral artery disease %	7	7,6	0.813
Atrial fibrillation %	26.4	12.7	**0.001**
Heart failure %	8.1	3.8	0.081
Renal insufficiency %	26	18.4	0.074
Urgent %	5	15.2	**0.000**

The primary outcome measures were 30-day, 90-day and long-term all-cause mortality. Secondary outcome measures were the 30-day reintervention rate and reintervention-free survival. A reintervention was defined as any adjunctive procedure due to an endoleak, access complication, thrombosis, kinking, abdominal wall dehiscence, anastomosis pseudoaneurysm, abscess or bleeding. Moreover, we evaluated aneurysm-related mortality (ARM), defined as death during the first 30 days after the primary or secondary intervention and death associated with aneurysm rupture or prothesis infection. The length of the hospital stay was also calculated.

### Imaging Parameters

Please see supplementary material.

### Statistics

The data were analysed with SPSS version 26 (SPSS Inc., Chicago, IL). Group comparisons were performed by using Student’s *t*-test, the chi-squared test, Fisher’s exact test and the Mann–Whitney *U* test.

The Cox regression method was used to calculate survival. Age, diabetes, hypercholesterolemia, hypertension, coronary artery disease, atrial fibrillation, peripheral artery disease, pulmonary disease and cerebrovascular diseases were examined as potential confounders and were tested in the Cox regression analyses. Smoking data were incomplete and were not taken in account. The Kaplan–Meier method was used to evaluate the reintervention rate according to treatment modality, and log-rank was used to evaluate the differences between the modalities; a *p* value of less than 0.05 was regarded as indicative of statistical significance.

## Results

A total of 416 patients met the inclusion criteria. Two hundred fifty-eight patients (62%) were treated endovascularly, while 158 (38%) underwent open repair. The majority of the patients were male (85.3% in the EVAR and 89.2% in the OSR group, *p* = 0.246). Patients treated endovascularly were markedly older than OSR patients (76.5 $\mp$ 7.3 vs. 68.8 $\mp$ 8.5, *p* =  < 0.000) and had a significantly higher rate of atrial fibrillation (26.4% vs. 12.7%, *p* = 0.001) and cardiovascular diseases (492% in the EVAR group vs. 31% in the OSR group, *p* = 0.000). There were no statistically significant differences between the groups in any other baseline characteristics (Table 2).

In the EVAR group, there were also significantly fewer urgently treated patients (5% in the EVAR vs. 15.2% in the OSR group, *p* = 0.000).

### Survival

The mean follow-up time was 6.1 $\mp$ 3.1 years in the EVAR group and 7.9 $\mp$ 3.8 years in the OSR group (*p* < 0.001). There were no intraoperative deaths in either group.

There was no significant difference in either 30-day mortality (1.6% in the EVAR group vs. 3.2% in the OSR group, *p* = 0.272) or 90-day mortality (2.7% vs. 4.4%, *p* = 0.346) between the groups. During the follow-up, 151 patients (58.5%) died in the EVAR group and 84 (53.2%) in the OSR group, with no significant difference between the groups (*p* = 0.284).

**Fig. 1:**
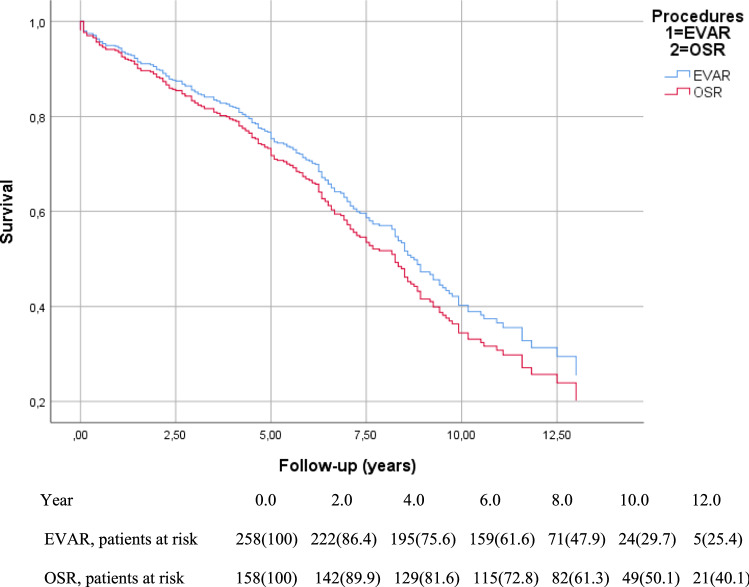
Cox regression survival curve for the whole cohort (*N* = 416)

In the Cox regression model, adjusting for common confounding factors, there was no significant difference in survival between the two treatment techniques (Fig. 1). Age, dyslipidemia, peripheral artery disease, congestive heart failure and cerebrovascular diseases remained the only factors influencing mortality, while compliance to the IFU did not influence mortality.

There were nine ruptures in the EVAR group (3.5%), of which seven died, one was converted, and two were treated successfully endovascularly. Of these patients, five presented with both type 1 and 2 endoleaks, one with endoleak type 1 and two with endoleak type 2. One patient did not adhere to the follow-up and eventually presented with a rupture due to endoleak type1, which was demonstrated at CTA. Taking into account early, procedure-related problems with open surgery, aneurysm-related mortality (ARM) was, however, similar in both groups (5.4% in the EVAR vs 4.4% in the OSR group, *p* = 0,652). However, the hospital stay was significantly shorter in the EVAR group, with a median of 3 days compared to 7 days among those treated with open repair (*p* < 0.001, Mann–Whitney Test).

### Reinterventions

The 30-day reintervention rate was significantly higher in the OSR group than the EVAR group (22 [13.9%] and 11 [4.3%], respectively, *p* < 0.001).

The most common complication in the OSR group was an abdominal fascia rupture, which occurred in 8 cases (5.1%), followed by laparotomy or exploration for haemostasis or a suspicion of bowel ischaemia (4.4%). In the EVAR group, the most common complication was a groin haemorrhage needing surgical revision, affecting 6 patients (2.3%).

**Fig. 2:**
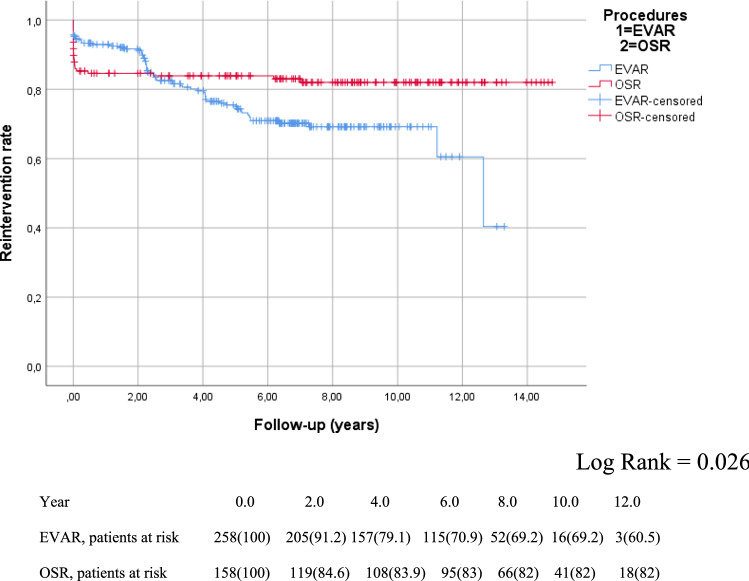
Kaplan–Meier curve for reintervention-free survival curve in the whole cohort (*N* = 416)

However, after the first 30 days, the reintervention-free survival curve for open surgery levelled out, while the decrease in the EVAR group continued throughout the follow-up period, indicating a significantly higher need for reinterventions (log-rank = 0.026; Fig. 2). After two years, there was a considerable increase in reinterventions in the EVAR group, probably due to our follow-up protocol, with the first CTA control scheduled after two years. For the most common Endoleak types in the EVAR population, refer to Table 3.

**Table 3:** Endoleak types in the EVAR population and in the EVAR non-IFU population

	EVAR	EVAR non-IFU
	*n* = 258	*n* = 114
Endoleak type I	16.3%	19.3%
Endoleak type Ia	7.4%	10.5%
Endoleak type Ib	8.9%	8.8%
Endoleak type II	29.1%	28.9%
Endoleak type III, endotension, migration and kinking	< 3%	3.5%

In the OSR group, the long-term complications were pseudoaneurysm of the anastomosis (2.5%), graft infection (2.5%) and limb occlusion (1.3%).

### Outside IFU Criteria Population

In the EVAR group, the non-adherence to IFU occurred in 114 patients (44%). These patients had similar baseline characteristics compared to the OSR group, with the exception of higher age (76,9 $\mp$ 7.2 vs. 68.8 $\mp$ 8.5, *p* < 0.001) and a higher presence of coronary artery diseases (48.2% vs. 31%, *p* = 0.005) in the EVAR group. Moreover, in the EVAR group, a trend towards more cerebrovascular diseases was also noted (*p* = 0.066). Further, in this subgroup analysis, there were more urgent cases in the OSR group (*p* < 0,001) (Table 4). The hospital stay was significantly shorter in the EVAR group, with a median of 3 days compared to 7 days among OSR patients (*p* < 0.001, Mann–Whitney Test).

**Table 4:** EVAR outside of IFU and OSR population baseline characteristics

	All		
	EVAR	OSR	p
	*N* = 114	*N* = 158	
Age, mean(years) SD	76.9	68.8	**0.000**
	SD 7.2	SD 8.5	
Sex, male %	81.6	89.2	0.079
Hypertension %	73.7	67.7	0.347
Dyslipidemia %	48.2	45.6	0.712
Coronary artery disease %	48.2	31	**0.005**
Diabetes %	21,9	17.1	0.350
Cerebrovascular disease %	17.5	9.5	0.066
Pulmonal disease %	19.3	17.7	0.753
Peripheral artery disease %	8.8	7.6	0.823
Atrial fibrillation %	20.2	12.7	0.129
Heart failure %	6.1	3.8	0.400
Renal insufficiency %	25.4	18.4	0.178
Urgent %	2,6	15.2	**0.000**

Table 5 shows the violations to IFU criteria in the EVAR group.

**Table 5:** Types of violations to IFU criteria and number of patients with one or more violation

IFU violations	No. (%)
Patients non-IFU, n	114 (44.2)
Neck length	14 (5.4)
Neck diameter	12 (4.7)
Neck angulation	13 (5.0)
Neck calcification	47 (18.2)
Neck thrombus	4 (1.6)
Neck conicity	23 (8.9)
Iliac diameter/length	18 (7.0)
1 violation	87 (32)
2 violations	18 (6.6)
3 violations	7 (2.6)
4 violations	2 (0.7)

There were altogether eight ruptures in the EVAR group (7%), while in the OSR none.

**Fig. 3:**
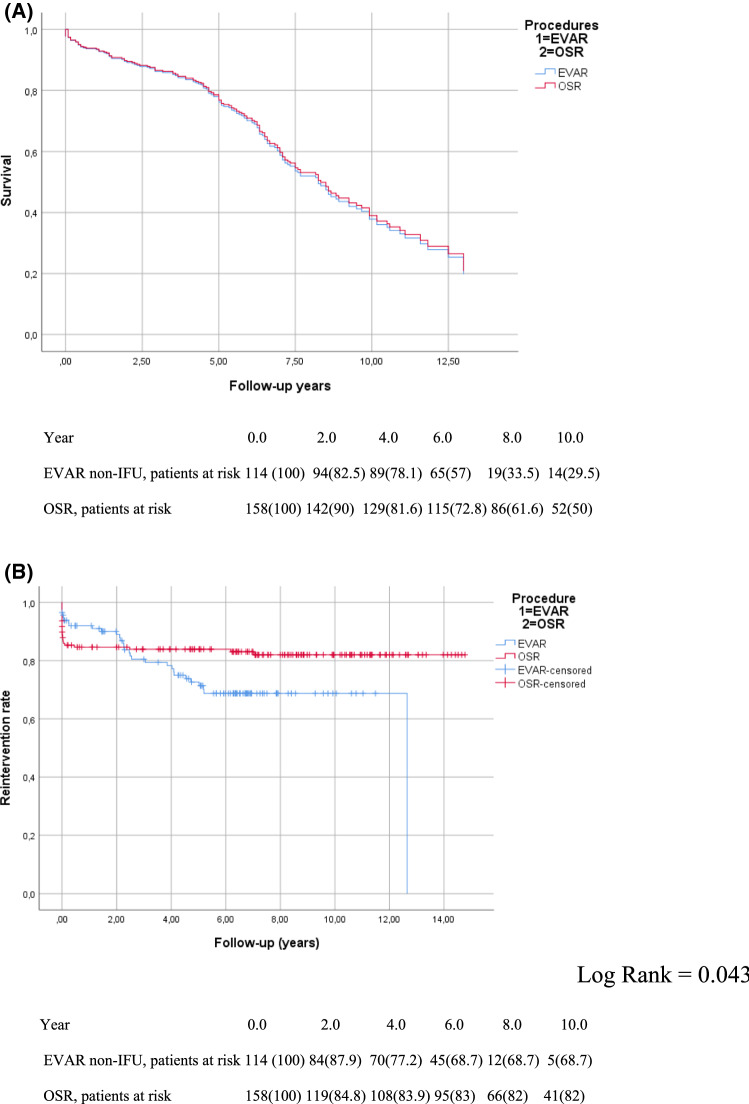
Outside-IFU population (*N* = 272): **A** Cox regression survival curve and **B** Kaplan–Meier reintervention-free survival curve

There was no significant difference in all-cause mortality at 30 or 90 days nor until the end of the follow-up (*p* = 1; *p* = 1 and *p* = 0.062, respectively). When analysed with the Cox regression model, there was no significant difference in overall survival between the EVAR and OSR groups. Age, hypercholesterolemia and cerebral artery disease were the only factors affecting survival (*p* < 0.001; *p* = 0.027; *p* < 0.001, respectively; Fig. 3a).

Aneurysm-related mortality was higher in the EVAR group (9.6%) than in the OSR group (4.4%), but the difference was not significant (*p* = 0.136). When confounding factors were evaluated with the Cox regression model, the procedure type did not affect mortality (*p* = 0.778) and age was the only significant factor (*p* = 0.005).

Also in this subpopulation, the OSR group had a significantly higher 30-day reintervention rate compared to the EVAR group (22 patients [13.9%] in the OSR group vs 4 [3.5%] in the EVAR group, *p* < 0.003). The Kaplan–Meier model for reintervention showed an inversion in the trend during the follow-up, with significantly more reinterventions being performed for EVAR patients (log-rank = 0.043; Fig. 3b).

## Discussion

In the current study, we could not find any difference in overall survival between the treatments after confounding factors were taken into account. Our results are in line with those of the DREAM trial, which showed no statistically significant difference in overall survival between treatment modalities after 12–15 years [12]. Furthermore, similar to their results, we found an increasing need for any reintervention in the EVAR group over time. Early reinterventions were, however, more common in the OSR group, a phenomenon which has been reported earlier [1, 2].

The need for more reinterventions during the follow-up did not correlate with higher mortality between EVAR and OSR. Geaorge A. Antoniou et al. was unable to show a significant difference in overall survival between treatment modalities, even though, also in their paper, the reintervention rate was higher in the EVAR group (13). In our study, the high number of reinterventions in the EVAR group could be partially explained by the fact that 114 (44.2%) patients did not adhere to IFU and that the criteria for reintervention changed over time. In particular, this concerns the treatment of type II endoleaks with a more active approach in the early years of the study. Aneurysm-related mortality was also similar between the two groups during the follow-up, which suggests that endoleaks can usually be properly treated.

In order to evaluate the effect of non-adherence to the IFU on outcome, a subanalysis was performed. Our hypothesis was that EVAR would perform less effectively than open repair. The topic is scarcely covered in the literature. Charbonneau et al. found a significant association between OSR and survival (HR 0.6; 95% CI, 0.4–0.9), showing higher overall long-term survival in the OSR group [10]. In contrast, we did not find any difference in overall survival between the groups. Moreover, in this subpopulation, ARM was similar between the groups, suggesting that both techniques are feasible. Contrary to the study of Charbonneau et al., we compared EVAR outside of IFU population with the whole OSR population as there are no widely accepted anatomical criteria for challenging AAA treated with open surgery.

The effect of non-adherence to IFU criteria in AAA treatment is not clear, and the available studies on this subject show controversial results. Some studies have described a relationship between IFU non-adherence and a poorer prognosis [14–17]. In contrast, other studies have demonstrated no difference in outcome independently of adherence to IFU criteria [18–20]. In a recent meta-analysis reporting on a total of 4498 patients treated with EVAR, Antoniou et al. found no difference in prognosis regardless of adherence to the IFU [20]. In line with these results is also the review by Oliveira et al., who compared the findings of 13 observational studies on EVAR performed outside the IFU and the results of the RCTs EVAR1, DREAM, ACE and OVER. This study showed no difference in mortality between the two populations. However, the follow-up was longer in the RCT group, which could have affected the findings [18]. In our study, the non-adherence to IFU also did not influence mortality. EVAR in non-IFU patients seems not to be inferior to OSR. The short-term reintervention rate is low, which makes the method appealing, especially for older patients with several comorbid conditions.

Nevertheless, it is interesting to notice that in the EVAR group we had nine ruptures, of which eight occurred in the outside of IFU group. This could be an indicator of worse outcome in the outside of IFU group as shown in our previous paper [17].

Our study has several limitations, being a non-randomized retrospective single-centre study. These factors could affect the results, especially regarding early and late mortality, because of unaccounted for confounders between the groups. Moreover, the EVAR group had more severe comorbidities, and the mean age was significantly higher. However, these biases serve to diminish the effectiveness of EVAR treatment compared to OSR. Furthermore, due to the relatively small study population, the subgroup analyses may be underpowered to detect small differences between subgroups.

## Conclusion

We did not find any statistically significant difference in overall survival or ARM between EVAR and OSR in long-term follow-up. In the short-term, reintervention rate was lower in the EVAR group, while during the follow-up, a significant growth in reinterventions in the EVAR population was encountered but without impact on overall survival or ARM This finding was also consistent independent of the adherence to IFU. Consequently, EVAR may be considered as an alternative also for patients outside IFU criteria for EVAR despite being surgical candidates. Further studies should be conducted to corroborate these results.

## Supplementary Information

Below is the link to the electronic supplementary material.

Supplementary file1 (DOCX 17 KB)
