# Supplementary material for: Endovascular Repair of Abdominal Aortic Aneurysms is a Valid Alternative to Open Repair also in Patients Treated Outside of Instructions for Use Criteria
Source: Cardiovasc Intervent Radiol. 2022 Nov 4;45(12):1765–73. doi: 10.1007/s00270-022-03297-7 (PMC9705501; doi:10.1007/s00270-022-03297-7)
Supplement: Supplementary file 1 — Supplementary file1 (DOCX 17 KB) [file 270_2022_3297_MOESM1_ESM.docx]

**Imaging parameters**

CT scans were obtained using two different multidetector scanners: General Electric LightSpeed 16-row (GE Healthcare, Milwaukee, WI, USA) and Philips Brilliance 64-row (Philips, Cleveland, OH, USA). The scanners were in equal use, and patients were not selected for a certain scanner. Abdominal aortic CT imaging was performed using the following parameters: 120 kV, 250 mAs, collimation 64 × 0.625 mm (64-row); or 120 kV, Auto MA (150-350 mAs), collimation 16 × 1.25 mm (16-row). Contiguous slices were reconstructed to the thickness of 1–3 mm over the entire scanning range. The contrast agent (Xenetix 350 mgI/mL, Aulnay-sous-Bois, France) was administered through an antecubital 18-G cannula with a double-piston power injector at a flow rate of 3 mL/s using 100 mL of contrast agent, followed by a 40-mL saline flush. Real-time bolus tracking was applied, and the acquisition was triggered when the contrast agent opacified the full diameter of the thoracoabdominal aorta. The acquisition was performed during deep-inspiration breath-hold.
